# The Expression Levels and Concentrations of PD-1 and PD-L1 Proteins in Septic Patients: A Systematic Review

**DOI:** 10.3390/diagnostics12082004

**Published:** 2022-08-19

**Authors:** Mutiara Indah Sari, Syafruddin Ilyas

**Affiliations:** 1 Department of Biochemistry, Faculty of Medicine, Universitas Sumatera Utara, Medan 20155, Indonesia; 2 Department of Biology, Faculty of Mathematics and Natural Sciences, Universitas Sumatera Utara, Medan 20155, Indonesia

**Keywords:** programmed death protein 1, programmed death ligand 1, sepsis, infection, immunosuppression

## Abstract

Sepsis is a series of life-threatening organ dysfunction caused by an impaired host response to infection. A large number of molecular studies of sepsis have revealed complex interactions between infectious agents and hosts that result in heterogeneous manifestations of sepsis. Sepsis can cause immunosuppression and increase the expression of checkpoint inhibitor molecules, including programmed death protein (PD-1) and programmed death ligand 1 (PD-L1), and thus PD-1 and PD-L1 are thought to be useful as diagnostic and prognostic tools for sepsis. PD-1 is an inhibitor of both adaptive and innate immune responses, and is expressed on activated T lymphocytes, natural killer (NK) cells, B lymphocytes, macrophages, dendritic cells (DCs), and monocytes, whereas PD-L1 is expressed on macrophages, some activated T and B cells, and mesenchymal stem cells as well as various non-hematopoietic cells. This systematic review aims to assess the PD-1 and PD-L1 protein expression levels and concentrations in septic and other infectious patients.

## 1. Introduction

Sepsis is a series of life-threatening organ dysfunction caused by an impaired host response to infection. One study states the overall incidence rate of sepsis is 288 per 100,000 person/year, whereas for severe sepsis it is 148 per 100,000 person/year [1,2]. Based on the data collected from high-income countries, the incidence of sepsis is estimated to be 31.5 million cases annually worldwide. Of these cases, 19.4 million are severe sepsis, and 5.3 million resulted in death each year [3].

The understanding of the molecular pathobiology and immunology of sepsis is constantly growing. It was previously assumed that the hemodynamic manifestations of sepsis were primarily associated with excessive immune responses to specific pathogens [3]. However, a large number of molecular studies of sepsis have revealed a much more complicated process that results in the heterogeneous manifestations of sepsis [4].

In sepsis, the first step of the host response to pathogens is the activation of innate immune cells, which mainly consist of neutrophils, macrophages, monocytes, and natural killer (NK) cells [5]. This occurs through the binding of pathogen-associated molecular patterns (PAMPs), such as bacterial endotoxin and fungal β-glucan to specific pattern recognition receptors, on these cells [4]. Another source of such interactions is damage-associated molecular patterns (DAMPs) which may be intracellular materials or molecules released from dead or damaged host cells, such as mitochondrial ATP and DNA [4,6]. They bind to specific receptors on monocytes and macrophages such as toll-like receptors (TLRs), C-type lectin receptors, NOD-like receptors (nucleotide-binding oligomerization domain) and RIG-1-like (retinoic acid-inducible gene 1) [4,6]. This results in the activation of intracellular signal transduction pathways leading to the transcription and release of proinflammatory cytokines such as TNFα, IL-1, and IL-6 [4].

A prolonged immunosuppression state often follows and replaces the early proinflammatory state of sepsis. The number of T cells (helper and cytotoxic) decreases as a result of apoptosis, and there is a decreased response to inflammatory cytokines [7]. Postmortem studies of deceased septic patients from the ICU showed a global depletion of CD4+ and CD8+ T cells, most of which were found mainly in lymphoid organs such as the spleen [8]. Studies have also shown decreased production of important cytokines such as IL-6 and TNF in response to endotoxins [9]. In septic patients, neutrophils are found to express fewer chemokine receptors, and there is decreased chemotaxis in response to IL-8 [10].

Sepsis-induced immunosuppression increases the expression of checkpoint inhibitor molecules, such as programmed death protein (PD-1), programmed death ligand 1 (PD-L1), B and T lymphocyte attenuator (BTLA), T cell membrane protein-3 (TIM-3), LAG3, etc. [11]. PD-1 is an inhibitor of both adaptive and innate immune responses and is expressed on activated T lymphocytes, NK cells, and B lymphocytes, macrophages, dendritic cells (DC), and monocytes, as well as in hematopoietic and non-hematopoietic cells. PD-1 plays opposite roles. On one side, PD-1 negatively regulates lymphocyte activation and function, reducing the harmful immune responses and maintaining immune tolerance. On the other hand, PD-1 interferes with the protective immune response and causes the development of malignant cells [12,13,14,15,16,17].

PD-1 ability to suppress T cell activation depends on the phosphorylation of immunoreceptor tyrosine-based switch motif (ITSM), which leads to phosphorylation of downstream effector molecules that play a negative regulatory role, and inhibits T cell proliferation and cytokine production [18]. The postmortem studies of septic patients revealed severe dysfunction in the spleen cells and the lungs also exhibit immunosuppressive effects, which may be due to self-programmed immune cell death induced by immunosuppression [19]. Inhibitory receptors expression on the surface of T cells is upregulated by chronic antigen stimulation, which is an important immunosuppressive mechanism in sepsis as well [20].

T cell receptor signaling and cytokines, such as IL-2, IL-7, and interferon type I (IFN), can induce PD-1 expression in T cells. PD-1 expression is especially increased in activated T cells and can be regulated within 24 h depending on the strength or concentration of the stimulus. The inhibitory effect of PD-1 is mediated by the PD-L1 and PD-L2 ligands [21].

PD-L1 is expressed on macrophages, some activated T and B cells, and mesenchymal stem cells as well as various non-hematopoietic cells, such as hepatocytes, vascular endothelial cells, epithelial cells, myocytes, pancreatic islet cells, and astrocytes [4,21]. Several studies have shown that increased expression of PD-1 and PD-L1 by T cells, monocytes, and neutrophils, and immunosuppression due to sepsis is one of the main risk factors for death in septic patients. Therefore, immunosuppression in sepsis provides a new understanding as well as a promising new target for treatment and prediction of its prognosis [13].

The immunoregulatory role played by PD-1 and PD-L1 during the inflammatory process in sepsis activates the PD-1 and PD-L1 pathway mechanisms to evade the body’s defenses (immunosuppression). Increased expression of PD-1 and PD-L1 molecules, and decreased CD28 molecules and CD86 induced immunosuppression in mice with sepsis. The expression of PD-1 on T lymphocytes, and CD14+ CD16+ monocytes showed a significant increase in septic patients along with the PD-L1 on CD14+ CD16+ monocytes [22]. Therefore, PD-1 may play an important role in the regulation of T lymphocytes and monocytes in septic patients, especially CD16+ monocytes associated with inflammation. Studies have also shown that PD-1 levels are found in patients and experimental animals with impaired immunity such as autoimmune diseases, chronic infections, and cancer [23,24,25].

In this review, we conduct a systematic review of studies of the expression levels of PD-1 and PD-L1 and the concentrations of sPD-1 and sPD-L1 proteins in patients with sepsis and other systemic infectious diseases. We hypothesize that published studies in their entirety would report the benefits of examining PD-1 and PD-L1 expression for both the diagnosis and prognosis of sepsis.

## 2. Methods

The study protocol was registered in PROSPERO (CRD42020210948) and was systematically reviewed following the Preferred Reporting Items for Systematic Reviews and Meta-analyses (PRISMA) guidelines [26].

A systematic online search was carried out from 15 April 2022 to 26 May 2022 in 4 search engines, namely PubMed, Springer Link, Wiley Online Library, and Proquest. The search was carried out using the keywords ((Sepsis OR Septicemia OR Pyemia OR Pyaemia OR Pyohemia OR Blood Poisoning OR Bloodstream Infection) AND (Healthy volunteer OR Healthy participant OR Healthy subject OR Human volunteer OR Normal volunteer OR Control Group) AND (PD 1 OR PD-1 OR PD1 OR Programmed Death 1 OR Programmed Cell Death Protein 1 OR Programmed Cell Death 1 Protein)) AND ((Sepsis OR Septicemia OR Pyemia OR Pyaemia OR Pyohemia OR Blood Poisoning OR Bloodstream Infection) AND (Healthy volunteer OR Healthy participant OR Healthy subject OR Human volunteer OR Normal volunteer OR Control Group) AND (PD-L1 OR PD L1 OR PDL1 OR Programmed Death Ligand 1 OR Programmed Cell Death 1 Ligand 1)). The data taken are the name of the first author, year of publication, research design, study output, and conclusion. After excluding the articles that did not match the title, abstract, and duplication, all articles that met the inclusion criteria were assessed for full text.

Title and abstract screening, data extraction, and risk assessment of bias were performed. Samples were taken based on PRISMA guidelines with the inclusion criteria of this study, namely research with septic patients or other infectious patients who develop sepsis and there is a healthy control group, with the expression level or protein level of PD-1 or PD-L1, and journals in English. The exclusion criteria are review articles, comments, case reports, dissertations or master’s theses, research conducted on animals, research older than 10 years, and research conducted on children.

The relevance of the study was determined using the inclusion criteria presented in Table 1. The study had to meet the following relevant criteria: population, intervention, comparison, and desired outcome. All screening stages use predefined questions—formulated following the PICO—to select the publications that cover the scope of the review.

## 3. Results

**Figure 1:**
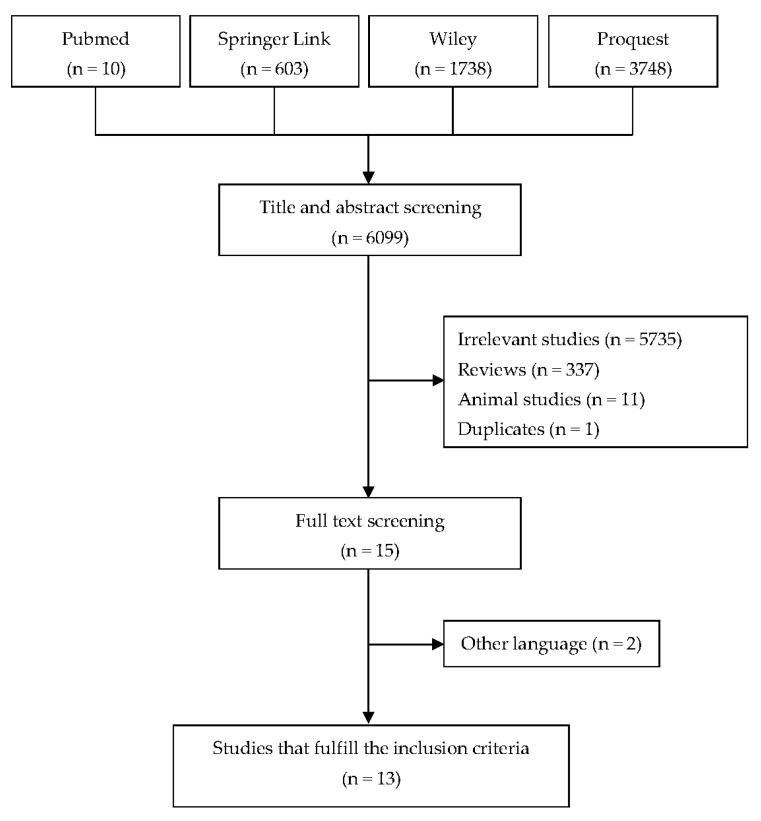
Flowchart for inclusion and exclusion.

The screening results of the search are presented in the PRISMA diagram as shown in Figure 1. By entering keywords and conducting a systematic search, we found 6099 studies across the search engines. In title and abstract screening, 6084 studies were excluded with the reasons: 5735 studies were irrelevant to this study, 337 studies were in the form of reviews, 11 studies were animal studies, and 1 study was a duplicate. After full text screening, 2 studies in a language other than English were excluded. The final result of the search is 13 studies that met the inclusion criteria in this systematic review, with 4 studies focused on PD-1, 2 studies focused on PD-L1, and 7 studies focused on PD-1 and PD-L1. The deadline for the search was 26 May 2022. The included studies are summarized in Table 2 and Table 3.

Of the 11 publications about PD-1, 9 found higher levels of PD-1 in septic patients, while 7 out of 9 publications about PD-L1 found higher levels of PD-L1 in septic patients. One of the two studies focuses on the PD-1 and PD-L1 in acute pancreatitis patients with and without infectious complications (IC), which includes infection, bacteremia, pneumonia, infected ascites, or urosepsis during admission and/or during the 90-day follow-up. This study finds significantly higher PD-1 and PD-L1 levels in acute pancreatitis patients with IC compared to non-IC on day 1 and day 3 (D1 *p* < 0.05; D3 *p* < 0.05). Meanwhile, the other study finds lower PD-1 levels in septic patients and no significant difference in the PD-L1 levels.

There are 7 publications studying the PD-1 and PD-L1 between survivors and non-survivors of sepsis, with 3 studies focused on PD-1, 1 study focused on PD-L1, and 3 studies focused on PD-1 and PD-L1. One study finds no significant difference in the sPD-1 levels between non-survivors and survivors of sepsis, but there is a significant increase in the sPD-L1 levels in the non-survivors group compared to the survivors group.

## 4. Discussion

As a member of the B7-CD28 superfamily, PD-1 plays an inhibitory role [39,40]. PD1 and PD-L1 can inhibit T and B cell functions such as cytokine production and cytotoxic activity and are thus important in immune regulation [41,42,43]. In addition, PD-1 also causes T cell “fatigue” so that T cells are dysfunctional, vulnerable to apoptosis, and unable to participate in an effective immune response, resulting in chronic viral infection [44]. Several studies have reported that the expression of PD-1 in lymphocytes and PD-L1 in monocytes causes immunosuppression during sepsis [34], leading to significantly increased levels in patients with sepsis [15,44,45].

PD-1 exists in two forms: a membrane-bound form and a soluble form (sPD-1). Like cytokines, sPD-1 circulates in the bloodstream to carry out its functions in the immune response and plays a major role in maintaining the balance of the PD-1/PD-L1 signaling pathway. Previous studies have shown that sPD-1 enhances T cell response through inhibition of the PD-1/PD-L1 signaling pathway and that excess sPD-1 can lead to immune imbalances in which auto-reactive T cells cannot function effectively; this can lead to pathological immune impairment [46]. Patients and experimental animals with autoimmune diseases, chronic viral infections, and cancer are found to have higher sPD-1 levels; however, correlations between sPD-1 levels and severe sepsis or septic shock are rarely reported [24,25,47].

This systematic review study strengthens the results of previous studies and existing theories, namely the increased PD-1 levels in septic patients compared to patients with non-infectious inflammation and healthy controls [27,28,29,31,32,33]. It was found that septic patients, especially those with severe sepsis and septic shock, have significantly higher levels of PD-1 expression in CD4+ or CD8+ T cells, PD-L1 expression in monocytes, sPD-1, and sPD-L1 levels [27,29,31,32,35,36,37], but there was no significant difference between the SIRS (Systemic Inflammatory Response Syndrome) group and healthy controls [34]. However, different results were obtained by Chang et al., 2014, which stated that increased PD-1 expression occurs only in CD8 T cells compared with non-septic patients, which is due to lymphocytes in septic patients having decreased IFN-γ and IL-2 and increased expression of PD-1 CD8 T cells [14]. This shows that both forms of PD-1 (membrane-bound and soluble form/sPD-1) are involved in the immune regulation of sepsis [29]. A secondary increase in sPD-1/sPD-L1 can occur when there is an increase in membrane-bound PD-1/PD-L1. When immune regulation is not controlled, the excessive sPD-1 can function as an antibody or a natural signaling inhibitor to block the PD-1/PD-L1 pathway, leading to impaired T cells activation and proliferation. sPD-1 can competitively bind to the PD-1 (PD-L1 and PD-L2) ligand, blocking the interaction on the surface of the cell membrane [24,48]. Therefore, a significantly increased level of sPD-1 may indicate more severe immunity impairment in patients with sepsis. This is supported by the results of research by Zhao et al., 2017 which stated that the more severe the sepsis is, the higher the levels of sPD-1/sPD-L1 will be. Increased sPD-1/sPD-L1 levels may indicate immune dysfunction in patients with severe sepsis or septic shock [34]. CD4+ PD-1+ expression levels and high heart rate are potential risk factors for sepsis [27].

Arens et al., 2016 found the opposite for PD-1 in septic patients in the long term. PD-1 levels were lower in septic patients compared to controls, while there were no significant differences in the PD-L1 levels between the two groups. However, BTLA receptor expression shows an inclination towards upregulation. As both are members of important negative regulators of T cell function, there is no clear conclusion. This may also be due to the small sample size of this study [30].

Among the septic patient groups, non-survivors showed higher levels of sPD-1 and sPD-L1 in peripheral blood, expression of PD-1 on CD4+ T cells and CD8+ T cells, and PD-L1 expression in monocytes than the survivors [31,32,35]. sPD-1 and sPD-L1 levels were positively correlated with the severity of sepsis. As the disease severity decreased in the survivor group, sPD-1 levels decreased in the first week. sPD-1 levels were an independent predictive factor for 28-day mortality on both day 1 and day 7. The prognostic evaluation of patients can be improved through monitoring sPD-1 levels during the first week of ICU stay [29].

This is supported by Huang et al., 2009 and Brahmamdam et al., 2010. A mouse model of sepsis shows a higher PD-1 expression level on the surface of peripheral blood macrophages and monocytes [47], and PD-1 antagonists significantly improved the survival of mice with severe sepsis or septic shock [49]. PD-1 overexpression in circulating T cells in septic patients is associated with decreased T cell proliferative capacity, increased secondary nosocomial infections, and death [47].

Different results were obtained by Liu et al., 2016. Non-survivors had significantly increased sPD-L1 levels compared to survivors (*p* < 0.05), but no statistical difference was found in serum sPD-1 levels between non-survivors and survivors (*p* > 0.05). Therefore, serum sPD-L1 may play a bigger role in sepsis compared to sPD-1 [35].

The percentage of CD4+ lymphocytes expressing PD-1 and CD14+ monocytes expressing PD-L1 on day 1 and day 3 was higher in acute pancreatitis patients compared to healthy controls and higher in the IC group of acute pancreatitis patients compared to non-IC [36]. This is supported by the theory that infectious factors play a role in the release of sPD-L1, which means that sPD-L1 is upregulated during infection and inflammation. For example, monocyte-derived DCs produced more sPD-L1 after in vitro experiments of LPS and TNF-α stimulation. Increased sPD-L1 was associated with increased levels of inflammation (CRP, PCT, WBC) and decreased immunological parameters (CD3+, CD4+ and CD8+). However, sPD-L1 levels decreased on the third day and decreased significantly on the seventh day compared to the first day of sepsis diagnosis. Compared to sepsis, septic patients had higher sPD-L1 levels compared to healthy controls, acute pancreatitis patients, and acute appendicitis, indicating that sPD-L1 is important for both identifying sepsis and assessing the severity of infection [33].

Liver cirrhosis (LC) patients show overexpression of PD-1 and PD-L1, which contribute to sepsis-associated immunosuppression and play an important role in bacterial infection [38]. Several other studies have also found increased levels of PD-1/PD-L1 and sPD-1/sPD-L1 in patients with liver diseases such as alcohol-related liver disease (ALD) [50] and chronic hepatitis B virus (CHBV) [46]. In this study, monocytic PD-L1 is excessive in LC patients, particularly those with severe complications of sepsis. Increased monocytic PD-L1 expression may be associated with immunosuppression and contribute to increased mortality in LC+ SS patients, though further investigation is needed for this hypothesis [38].

Jia et al., 2016 showed that both T2DM (type 2 diabetes mellitus) patients and patients with severe sepsis had decreased immunity. Compared to healthy controls, PD-1 levels in peripheral blood T lymphocytes increased in both T2DM and severe sepsis patients. Patients with severe sepsis also showed higher PD-1 levels than T2DM patients. Patients with severe sepsis and T2DM had similar T cell PD-1+ CD4+ and T cell PD-1+ CD8+ expression and mortality rates in patients with only severe sepsis. This study also showed that T2DM increased neither the 28-day mortality in patients with severe sepsis nor the expression of PD-1 on T cells. Non-survivors also had higher PD-1 levels on T cells which may be associated with more severe immunosuppression. The SS group and the SS+ T2DM group did not show a significant difference in the expression of PD-1 in T cells and instead showed a slight increase compared to healthy controls, likely because the increase in PD-1 in T2DM was less pronounced compared to severe sepsis. Septic patients, with or without T2DM, still had increased expression levels and concentrations of PD-1 [28].

## 5. Conclusions

Septic patients, especially those with severe sepsis and septic shock, had significantly higher PD-1 expression levels on CD4+ or CD8+ T cells, PD-L1 expression on monocytes, sPD-1, and sPD-L1 levels compared to patients with non-septic infections, non-infectious inflammation, and a healthy control group. Meanwhile, non-survivors showed higher levels of sPD-1 and sPD-L1 in peripheral blood, expression of PD-1 on CD4+ T cells and CD8+ T cells, and PD-L1 expression in monocytes than survivors. Blocking PD-1 by using inhibitory antibodies has been shown to restore T cell function, increase the antiviral response in T cells, decrease viral load in sepsis, and decrease apoptosis.

## Figures and Tables

**Table 1:** List of questions used for inclusion and exclusion of studies over the title and abstract, as well as the full text.

Screening Stage	Questions	Results
Title and abstract screening	Is this study focused on sepsis or other systemic infections on adults? Does this study show the expression levels of PD-1 or PD-L1 or the concentrations of sPD-1 or sPD-L1? Is this study published within the last 10 years? Is the study design case control, cohort, cross sectional, randomized controlled trial (RCT), or meta-analysis?	Study included if all questions are answered with ‘yes’
Full text screening	Does this study have a control group as a comparison? Is this study in English? Is there the full access to this study? Does this study have clear diagnostic criteria for sepsis? Are there exclusion criteria for the septic patients in this study (such as immunodeficiency)?	Study included if all questions are answered with ‘yes’

**Table 2:** Study characteristics of expression level and concentration of PD-1 protein in septic patients.

Reference	Study Design	n	Patients	Outcome
Gao et al., 2015 [27]	Cross sectional	367	182 septic patients (54 mild sepsis, 46 severe sepsis, 82 septic shock), 185 healthy controls	Significantly higher expression levels of CD4+ CTLA-4+, CD4+ PD-1+ and CD8+ PD-1+ on all sepsis subgroups than the control group (*p* < 0.05) Expression level of CD4+ PD-1+ is proven to be risk factors with statistical significances by logistic regression analysis (*p* = 0.003)
Jia et al., 2016 [28]	Prospective and observational study	295	80 T2DM patients without sepsis, 88 severe septic (SS) patients without T2DM (29 non-survivors), 77 SS patients + T2DM (24 non-survivors), 50 healthy controls	Significantly increased percentage of PD-1+ CD4+ T cells and PD-1+ CD8+ T cells in the T2DM, SS and SS+ T2DM groups compared to the healthy control group and highest levels in the SS+ T2DM and SS group, but no significant difference between the SS+ T2DM group and the SS group Significantly higher percentage of PD-1+ CD4+ T cells and PD-1+ CD8+ T cells, mean fluorescence intensity (MFI) PD-1+ CD4+ T cells and PD-1+ CD8+ T cells in non-survivors than survivors among SS patients with or without T2DM (*p* < 0.05), but in the survivor or non-survivor group, no significant difference between those with T2DM and those without T2DM (*p* > 0.05)
Zhao et al., 2019 [29]	Cohort study	157	72 severe septic patients, 40 septic shock patients, 45 healthy controls; 112 septic patients were separated into 73 survivors and 39 non-survivors	Significantly higher levels of sPD-1, MFI value of PD-1 on CD4+ and CD8+ T cells in the patients with severe sepsis or septic shock on day 1 of ICU admission than healthy controls (*p* < 0.001) Significantly higher sPD-1 in non-survivors than survivors (*p* < 0.05) Significantly higher percentages and MFI values of PD-1 on CD4+and CD8+ T cells in non-survivors than survivors (*p* < 0.05) No significant differences in the expression of PD-1 on CD4+ and CD8+ T cells and the expression of PD-L1 on monocytes between survivors and non-survivors between day 1 and day 7
Arens et al., 2016 [30]	Cross sectional	16	8 septic patients, 8 healthy controls	In CD4+ cells from septic patients, PD-1 receptor density appears to be downregulated No substantial changes in CD8+ cells PD-1 receptor density in septic patients
Chang et al., 2014 [15]	Cross sectional	58	43 septic patients, 15 non-septic patients	Increased expression of CD8 but not CD4 PD-1 in septic patients compared to non-septic patients Increased CD8 T cell expression of PD-1 over time in the ICU as PD-L1 decreased
Pan et al., 2015 [31]	Observational/clinical study	86	56 septic shock patients, 30 healthy controls	Significantly greater expressions of PD-1 in the CD4+ T cells in septic shock patients compared to healthy volunteers Much higher expressions of PD-1 expressions on monocytes in septic shock patients compared to healthy volunteers
Shao et al., 2016 [32]	Prospective cohort study	164	59 septic patients, 76 septic shock patients (49 survivors, 27 non-survivors), 29 healthy controls	Significantly increased percentage of PD-1/CD4+ T cells and PD-1/CD8+ T cells in patients with sepsis and septic shock (*p* < 0.05) compared to healthy controls, and was significantly higher in non-survivors than survivors of septic shock (*p* < 0.05)
Pan et al., 2017 [33]	Prospective study	95	63 acute pancreatitis (25 with IC, 38 non-IC), 32 healthy controls	Significantly higher percentage of CD4+ lymphocytes expressing PD-1 on day 1 and day 3 in the group of acute pancreatitis patients with IC compared to non-IC (D1 *p* < 0.05; D3 *p* < 0.05) The percentage of CD14+ monocytes expressing PD-L1 was more closely related to IC in acute pancreatitis than with PD-1 expression in CD4+ lymphocytes
Zhao et al., 2017 [34]	Prospective cohort study	655	595 emergency department (ED) patients (130 SIRS, 276 sepsis, 121 severe sepsis, 68 septic shock; 465 septic patients were separated into 349 survivors and 116 non-survivors), 60 healthy controls	No significant difference in sPD-1 levels between the SIRS group and the healthy control group (*p* = 0.462) Higher sPD-1 levels in the sepsis group compared to SIRS and healthy control group (*p* < 0.05) Significant difference in pairwise comparison of sPD-1 levels in the sepsis, severe sepsis, and septic shock groups As the severity of sepsis increases, sPD-1 levels gradually increase. Higher sPD-1 levels in non-survivor group than the survivor group sPD-1 showed the highest specificity (91.8%) for predicting 28-day mortality of septic patients Lower probability of survival at 28 days in septic patients with sPD-1 levels higher than 2.38 ng/mL than septic patients with lower sPD-1 levels (*p* < 0.001)
Liu et al., 2016 [35]	Observation study	120	91 septic patients (53 survivors and 38 non-survivors), 29 healthy controls	Significantly increased sPD-1 levels in septic patients compared to healthy controls (*p* = 0.000) No statistical difference in serum sPD-1 levels between non-survivors and survivors (*p* > 0.05)
Liu et al., 2017 [36]	Observation study	139	78 severe septic patients, 40 septic shock patients, 21 healthy controls; 118 septic patients were separated into 60 survivors and 58 non-survivors	Significantly increased PD-1 expression in regulatory T cells (Tregs) in septic patients compared to healthy controls, but no significant difference between the severe sepsis group and the septic shock group Upregulation of PD-1 expression on CD4+ T cells in septic patients compared to healthy controls, but no significant difference between the severe sepsis and septic shock groups. Significantly higher PD-1 expression in Treg and CD4+ T cells in non-survivors than survivors (*p* < 0.001 and *p* = 0.001, respectively)

**Table 3:** Study characteristics of expression level and concentration of PD-L1 protein in septic patients.

**Reference**	**Study Design**	**n**	**Patients**	**Outcome**
Sun et al., 2021 [37]	Cross sectional	156	64 septic patients, 29 appendicitis patients, 33 acute pancreatitis patients, 30 healthy controls	Increased sPD-L1 levels on day 1 in septic patients compared to healthy controls, acute pancreatitis, and acute appendicitis patients Significant positive correlation between sPD-L1 with inflammatory markers such as CRP (*p* = 0.001), PCT (*p* = 0.001), WBC (*p* = 0.012) Significant negative correlation between sPD-L1 with immunological functional parameters such as CD3+ counts (*p* < 0.001), CD4+ counts (*p* < 0.001) and CD8+ counts (*p* < 0.001) Decreased sPD-L1 levels on the third day and a significant decrease on the seventh day compared to the first day of sepsis diagnosis (*p* < 0.01) No correlation between sPD-L1 and lymphocytes, levels of IgM, IgG, and IgA
Zhao et al., 2019 [29]	Cohort study	157	72 severe septic patients, 40 septic shock patients, 45 healthy patients	Increased PD-L1 expression in patients with severe sepsis or septic shock. Significantly higher levels of sPD-L1, MFI value of PD-L1 on monocytes in the patients with severe sepsis or septic shock on day 1 of ICU admission than healthy controls (*p* < 0.001) Significantly different percentages of PD-L1 on monocytes between the severe sepsis and septic shock groups (*p* < 0.05) Significantly higher percentages and MFI of PD-L1+ on monocytes in non-survivors than survivors (*p* < 0.05) Positive correlation between sPD-L1 levels with the severity of sepsis
Arens et al., 2016 [30]	Cross sectional	16	8 septic patients, 8 healthy controls	No substantial difference for TLR4 and PD-L1 between the two groups
Chang et al., 2014 [15]	Cross sectional	58	43 septic patients, 15 non-septic patients	Over two-fold increase in the percentage of PD-L1-expressing monocytes in septic versus non-septic patients Monocytic PD-L1 from septic patients remained elevated but did not change over time Increased CD8 T cell expression of PD-1 over time in the ICU as PD-L1 decreased
Pan et al., 2015 [31]	Observational/clinical study	86	56 septic shock patients, 30 healthy controls	Significantly greater expressions of PD-L1 in the CD4+ T cells in septic shock patients compared to healthy volunteers Much higher expressions of PD-L1 expressions on monocytes in septic shock patients compared to healthy volunteers
Shao et al., 2016 [32]	Prospective cohort study	164	59 septic patients, 76 septic shock patients (49 survivors, 27 non-survivors), 29 healthy controls	Significantly increased percentage of monocytes expressing PD-L1 in patients with sepsis and septic shock (*p* < 0.05) compared to healthy controls, and was significantly higher in non-survivors than survivors of septic shock (*p* < 0.05)
Lu et al., 2021 [38]	Observational pilot study	117	30 liver cirrhosis (LC) patients, 70 liver cirrhosis + severe sepsis (LC + SS) patients (59 with SS-induced acute on chronic liver failure (ACLF); 37 survivors, 22 non-survivors), 17 healthy controls (HC)	Higher percentage of monocytic PD-L1 in the LC + SS group compared to the LC group (*p* = 0.046) and HC group (*p* < 0.001) and lower in the HC group compared to the LC group (*p* = 0.018) [HC < LC < LC + SS] Higher mean fluorescence intensity (MFI) of monocytic PD-L1 in the LC+ SS group compared to the LC (*p* = 0.046) and HC (*p* = 0.009) groups. Significantly higher monocytic PD-L1 percentage (*p* = 0.004) and MFI (*p* = 0.008) in the deceased group than the living group
Pan et al., 2017 [33]	Prospective study	64	63 acute pancreatitis (25 with IC, 38 non-IC), 32 healthy controls	Significantly higher percentage of CD14+ monocytes expressing PD-L1 on day 1 and day 3 in the group of acute pancreatitis patients with IC compared to non-IC (D1 *p* < 0.05; D3 *p* < 0.05) The percentage of CD14+ monocytes expressing PD-L1 was more closely related to IC in acute pancreatitis than with PD-1 expression in CD4+ lymphocytes
Liu et al., 2016 [35]	Observation study	120	91 septic patients (53 survivors and 38 non-survivors), 29 healthy controls.	Significantly increased serum sPD-L1 levels in septic patients compared to healthy controls (*p* = 0.000) Significantly increased serum sPD-L1 levels in non-survivors compared with survivors (*p* < 0.05)

## Data Availability

Not applicable.
