# Correction: Sari, M.I.; Ilyas, S. The Expression Levels and Concentrations of PD-1 and PD-L1 Proteins in Septic Patients: A Systematic Review. *Diagnostics* 2022, *12*, 2004

**DOI:** 10.3390/diagnostics13091555

**Published:** 2023-04-26

**Authors:** Mutiara Indah Sari, Syafruddin Ilyas

**Affiliations:** 1 Department of Biochemistry, Faculty of Medicine, Universitas Sumatera Utara, Medan 20155, Indonesia; 2 Department of Biology, Faculty of Mathematics and Natural Sciences, Universitas Sumatera Utara, Medan 20155, Indonesia

There was an error in the original publication [1]. We misunderstood how PROSPERO registration numbers worked then, so we thought that any number available on the website could work. We have removed the PROSPERO number (CRD42020210948) from the article.

A correction has been made to Methods, Paragraph 1 on the first line:

“The study was systematically reviewed following the Preferred Reporting Items for Systematic Reviews and Meta-analyses (PRISMA) guidelines [26].”

The authors state that the scientific conclusions are unaffected. This correction was approved by the Academic Editor. The original publication has also been updated.
